# Author Correction: The Adipocyte Na/K-ATPase Oxidant Amplification Loop is the Central Regulator of Western Diet-Induced Obesity and Associated Comorbidities

**DOI:** 10.1038/s41598-020-75948-z

**Published:** 2020-11-05

**Authors:** Rebecca D. Pratt, Cameron Brickman, Athar Nawab, Cameron Cottrill, Brian Snoad, Hari Vishal Lakhani, Austin Jelcick, Brandon Henderson, Niharika N. Bhardwaj, Juan R. Sanabria, Jiang Liu, Zijian Xie, Nader G. Abraham, Joseph I. Shapiro, Komal Sodhi

**Affiliations:** 1 Departments of Medicine, Surgery, Biomedical Sciences, and Healthcare Informatics Program, Joan C. Edwards School of Medicine, Marshall University, Huntington, WV USA; 2 Vector Builder Incorporated, Shenandoah, TX 77384 USA; 3 Department of Medicine, New York Medical College, Valhalla, NY 10595 USA

Correction to: *Scientific Reports* https://doi.org/10.1038/s41598-019-44350-9, published online 28 June 2019

The original version of this Article contained an error. A Data Availability section was originally not included – it now appears as below:

## Data Availability

Raw data generated for this study has been deposited at an online open access repository “Figshare” and can be accessed via this link: https://figshare.com/authors/Rebecca_Pratt/8485863

Following are the individual DOIs for raw data for each tissue:

Adipose: https://doi.org/10.6084/m9.figshare.11891760

Liver: https://doi.org/10.6084/m9.figshare.11892111

Brain: https://doi.org/10.6084/m9.figshare.11892279

In addition, there were errors in Figure S2 of the updated supplementary file, where the CTR images for Green Fluorescence and Red Fluorescence corresponding to DAPI were incorrect. In the revised Supplementary Figure 2, the representative cluster for images (green fluorescence and red fluorescence) within each group now correctly identifies with their corresponding DAPI images. These corrections do not affect the findings of the original article. Specifically, the following representative images have been replaced in Supplementary Figure 2: Green and Red Fluorescence images of Liver tissue for Control and WD groups; Green and Red Fluorescence images of Brain tissue for Control, WD and WD-Lenti-Adiponectin-GFP groups.

These errors have now been corrected in the HTML and PDF versions of the Article, and in the accompanying Supplementary Information file.

